# Recent advances in CAR‐T cell therapy for acute myeloid leukaemia

**DOI:** 10.1111/jcmm.18369

**Published:** 2024-05-07

**Authors:** Chi Gao, Xin Li, Yao Xu, Tongcun Zhang, Haichuan Zhu, Di Yao

**Affiliations:** ^1^ College of Life Science and Health Wuhan University of Science and Technology Wuhan China; ^2^ College of Biotechnology Tianjin University of Science and Technology Tianjin China; ^3^ Institute of Biology and Medicine Wuhan University of Science and Technology Wuhan China

**Keywords:** acute myeloid leukaemia, chimeric antigen receptor T cells, scFv, target antigen

## Abstract

Acute myeloid leukaemia (AML) is a fatal and refractory haematologic cancer that primarily affects adults. It interferes with bone marrow cell proliferation. Patients have a 5 years survival rate of less than 30% despite the availability of several treatments, including chemotherapy, allogeneic haematopoietic stem cell transplantation (Allo‐HSCT), and receptor antagonist drugs. Allo‐HSCT is the mainstay of acute myeloid leukaemia treatment. Although it does work, there are severe side effects, such as graft‐versus‐host disease (GVHD). In recent years, chimeric antigen receptor (CAR)‐T cell therapies have made significant progress in the treatment of cancer. These engineered T cells can locate and recognize tumour cells in vivo and release a large number of effectors through immune action to effectively kill tumour cells. CAR‐T cells are among the most effective cancer treatments because of this property. CAR‐T cells have demonstrated positive therapeutic results in the treatment of acute myeloid leukaemia, according to numerous clinical investigations. This review highlights recent progress in new targets for AML immunotherapy, and the limitations, and difficulties of CAR‐T therapy for AML.

## 1 INTRODUCTION

Acute myelocytic leukaemia (AML) is a clonal, malignant, proliferative condition that affects the myeloid primitive cells of the haematopoietic system. Acute myeloid leukaemia is a clonal expansion in bone marrow and is highly malignant. Haematopoietic progenitor cells that have experienced malignant transformation at different phases of the differentiation and development of normal myeloid cells can turn into diverse illnesses.^1^ Allogeneic haematopoietic stem cell transplantation (Allo‐HSCT) is now the only option for patients with high‐risk or recurrent AML, however, it requires a matched donor.^2^ The therapeutic efficiency of Allo‐HSCT is driven by the graft‐versus‐leukaemia phenomenon, which occurs when donor T cells recognize invading pathogens on the host's CAR‐T cells in the haematopoietic tissue of bone marrow malignancies and subsequently mediate tumour elimination. This GvL phenomenon illustrates how bone marrow responds to T‐lymphocytes. Unfortunately, graft‐versus‐host disease (GVHD), which has a high morbidity and mortality rate, can be caused by donor immune effector cells attacking healthy organs. Numerous immunotherapies, such as checkpoint inhibitors, antibody‐drug conjugates, T‐cell splicing agents, and cellular treatments, have been launched in clinical trials as our knowledge of the leukaemia microenvironment and immune milieu has grown. In select subgroups of AML patients with immune‐enriched characteristics, checkpoint inhibitors have had some success.^3^ Early findings with fletuzumab (CD3‐CD123 bispecific antibody) and Magrolimab (anti‐CD47 monoclonal antibody) are anticipated following the approval of a modest split dosage of Gemtuzumab ozogamici (gituzumab), paving the way for AML immune‐based treatments.^4^, ^5^ In an innovative form of immunotherapy known as chimeric antigen receptor (CAR) T cells, naturally existing T cells are genetically altered to produce specific CARs that allow them to recognize and destroy tumour cells. After many years of preclinical and clinical study, CAR‐T cells are currently acknowledged as the best therapeutic approach. CAR‐T cell treatment has been effective in treating B‐cell lymphoma and acute lymphoblastic leukaemia (ALL).^6^, ^7^, ^8^, ^9^, ^10^, ^11^, ^12^, ^13^, ^14^, ^15^, ^16^ Additionally, CAR‐T cell treatment for AML is presently being investigated. Unfortunately, the lack of suitable cancer antigens prevented the development of CAR‐T cells in AML. To minimize the side effects of CAR‐T cell treatment, the optimal antigen should be produced primarily on malignant tumour cells. Finding the best target for this illness can be challenging because the bulk of the antigens produced by AML cells are also present in healthy haematopoietic cells.^17^ The development of various CAR‐T cell therapy targets for AML, as well as the drawbacks and difficulties of CAR‐T for AML, will be the main topics of this review.

## 2 STRUCTURE OF CAR

### 2.1 Evolution of CAR design

**FIGURE 1:**
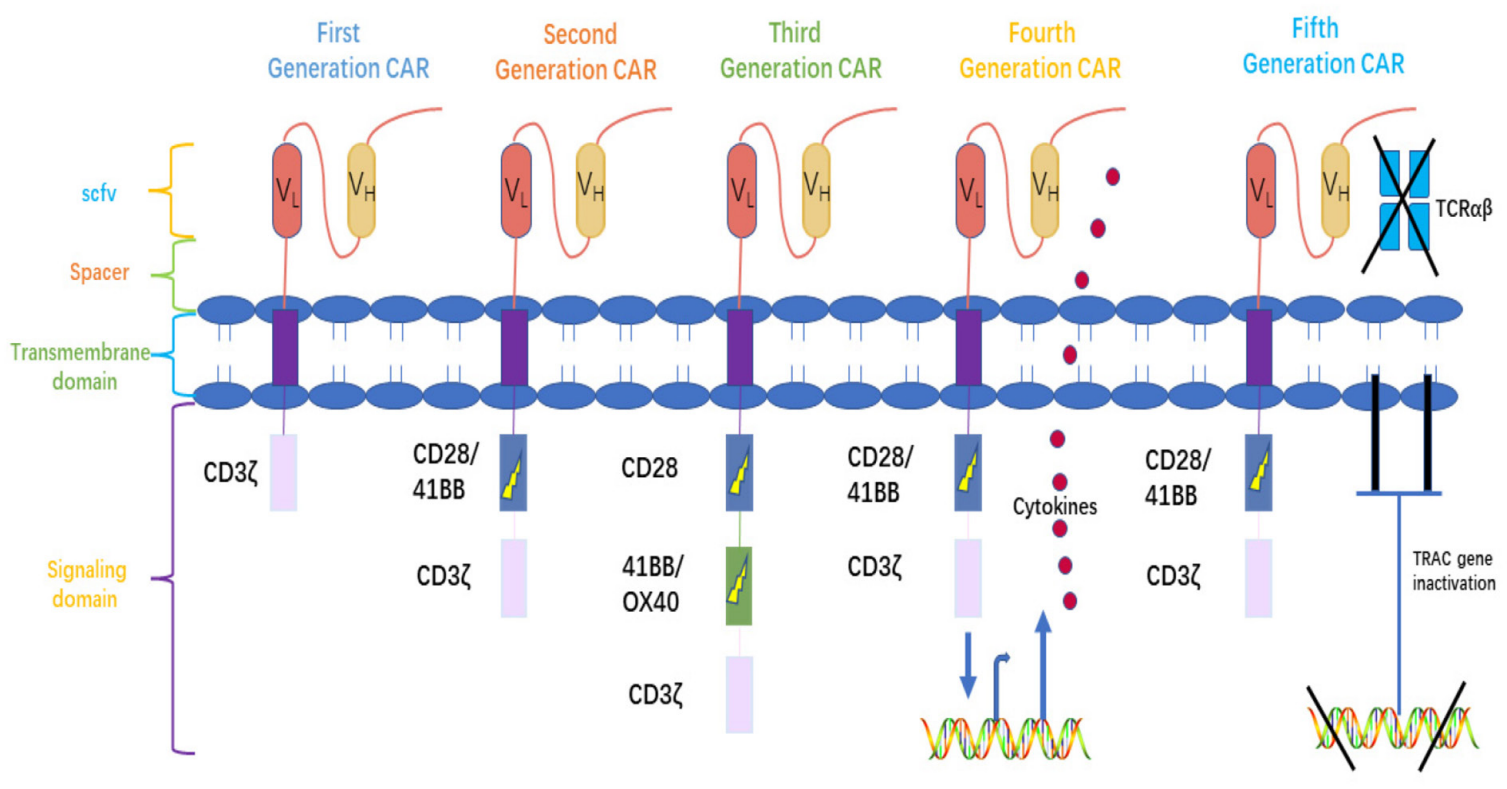
CAR structure and its evolution.

CAR is an artificial receptor molecule created by genetic engineering technology that confers specificity to immune effector cells against a target antigenic epitope, thereby enhancing T‐lymphocyte recognition of antigenic signals and activation.^18^ The CAR structure consists of an extracellular binding region that is usually the single‐chain fragment variable (scFv) region, a hinge, a transmembrane domain, and an intracellular signalling domain.^19^, ^20^ A typical intracellular signalling region contains a CD3 activation domain and a co‐stimulatory proliferative signalling domain. T cells can behave as tumour‐killing cells.^21^ Cytotoxic T cells (CTLs) are key effector cells of the anti‐tumor immune response, specifically recognizing tumour cells when the body is stimulated by antigens through the interaction of the T cell receptor (TCR) with the major histocompatibility complex. The CAR has undergone an evolution spanning five successive generations thus far. Zelig Eshhar developed the first‐generation CAR in 1989 by splicing the heavy and light chain variable sections of the monoclonal antibody into T cells together with a fixed percentage of the TCR.^22^ The first generation of CARs only contained one intracellular region, primarily CD3, which caused T cells to become activated but had a limited ability to wake up dormant T cells and to maintain a sustained T cell response or cytokine release.^23^ Second‐generation CARs include not only the CD3ζ‐activated region but also a single co‐stimulatory molecule, CD28 or 4‐BB, and in the third‐generation CARs, a second co‐stimulatory molecule, OX‐40, are added,^24^ and these additional stimulatory molecules enhance T‐cell activation, persistence and proliferation. With the addition of CAR‐inducible genes that encode diverse cytokines and mediators to promote CAR activation, fourth‐generation CARs are built based on second‐generation CARs, and T cells transduced with fourth‐generation CARs can be employed for universal cytokine‐mediated killing.^25^ Significantly improved therapeutic efficacy and decreased systemic toxicity have been reported in preclinical studies. Fifth‐generation CAR‐T cells, also known as next‐generation CAR‐T cells, are currently being studied. The next‐generation CAR‐T cells include one more functional element in CAR‐T structure compared with previous versions. A truncated intracellular domain of cytokine receptors with a binding site for transcription factors STAT3/5 was integrated into the CAR‐T intracellular structure (Figure 1). The addition of extra functional domains helped the CAR‐T cells to better achieve their goals.^26^

#### 2.1.1 Single‐chain fragment variable (scFv)

The antibody heavy chain variable region and its light chain variable region were joined together to form the scFv, which serves as the CAR's antigen‐binding element, which can specifically recognize antigens on the surface of the target cell.^27^ Because of the high specificity of this kind of antigen–antibody interaction, scFv is pivotal for the CAR‐T cells to execute their precise attack on cancer cells.^28^

#### 2.1.2 Extracellular spacer region

Also known as the ‘hinge region’, it provides flexibility and is typically made up of immunoglobulin‐based components like CH2, CH3 and CH2CH3.^29^ Hinges derived from native T cell molecules, such as CD8 and CD28 hinge regions, were approved for clinical CAR‐T cell therapies.

#### 2.1.3 Transmembrane structural domains

The transmembrane domain linked the extracellular hinge to the intracellular signalling domain.^30^ The majority of transmembrane structures are derived from CD3ζ, CD4, CD8 or CD28 molecules.^31^ Signals will be transduced via this domain from extracellular to intercellular, which can significantly affect the killing efficiency of CAR‐T cells.

#### 2.1.4 Intracellular signalling domains

The cytoplasmic structure of CAR‐T was composed of signalling domains necessary for the activation of T cells. Co‐stimulation domains were added to the cytoplasmic structure in the later‐developed CAR‐T version. CD3 structural domain is the primary signalling region within the cell, while other co‐stimulatory molecules like CD28, 4‐1BB, or OX40 may also be present.^32^ Nevertheless, they do not function as effectively as CD3ζ does. Other immunoreceptor tyrosine‐activated motifs (ITAMs), such as the IgE‐structural domain of the Fc receptor, have also been suggested as intracellular activating structural domains.^33^

## 3 CAR‐T CELL THERAPY

**FIGURE 2:**
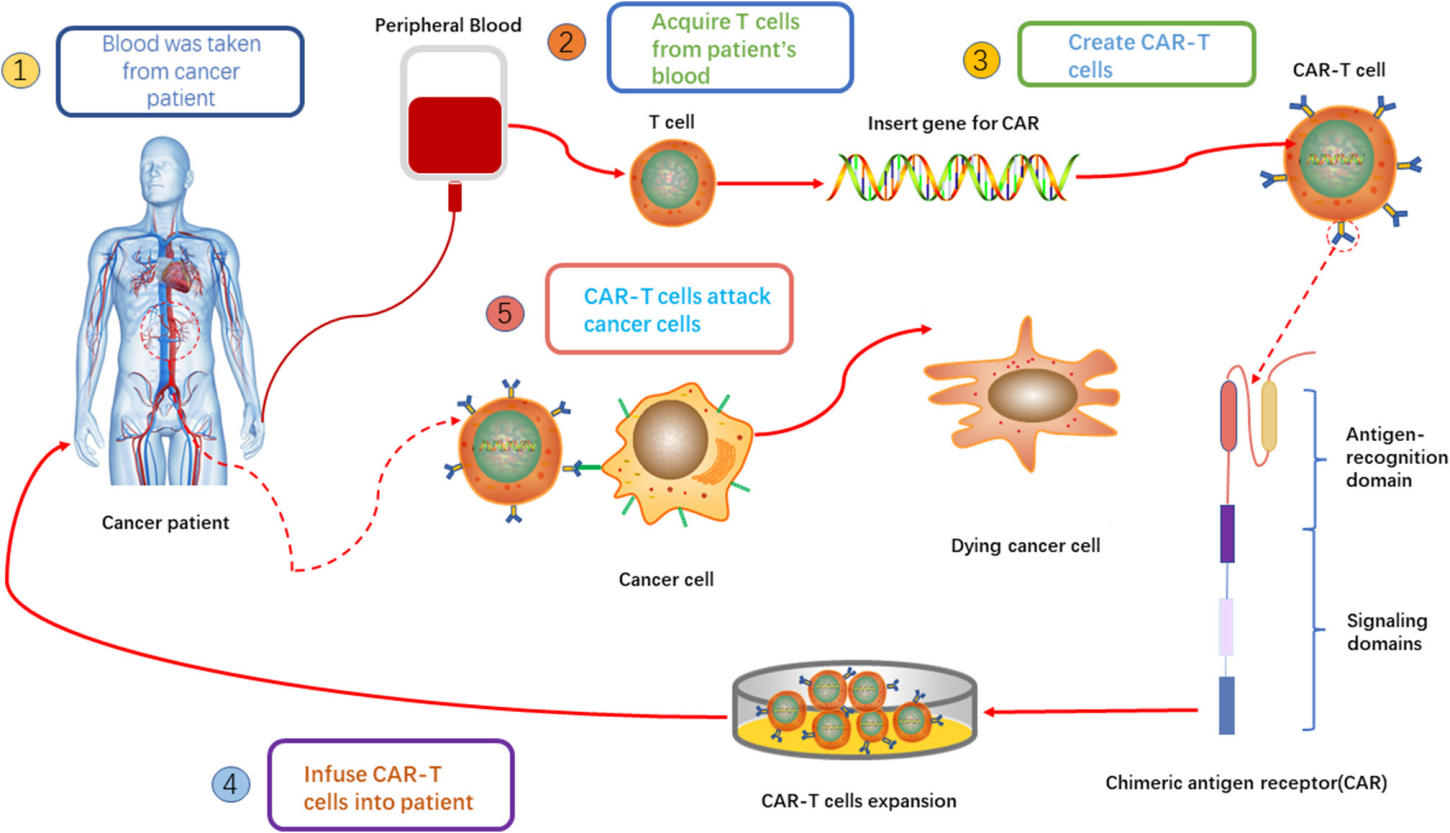
CAR‐T cell therapy flow.

T cells are separated from the patient's peripheral blood mononuclear cells (PBMC). These gathered T cells are stimulated using magnetic beads or exogenous cytokines. Then, using mRNA electroporation, gene editing techniques, liposomes, lentiviruses or retroviruses, CAR‐expressed genes are transferred into these proliferated T cells to drive the cells to detect the tumour. These CAR‐T cells are multiplied and then put back into the patient while they are being watched^34^ (Figure 2).

## 4 HISTORICAL PROGRESS OF CAR‐T CELLS

**FIGURE 3:**
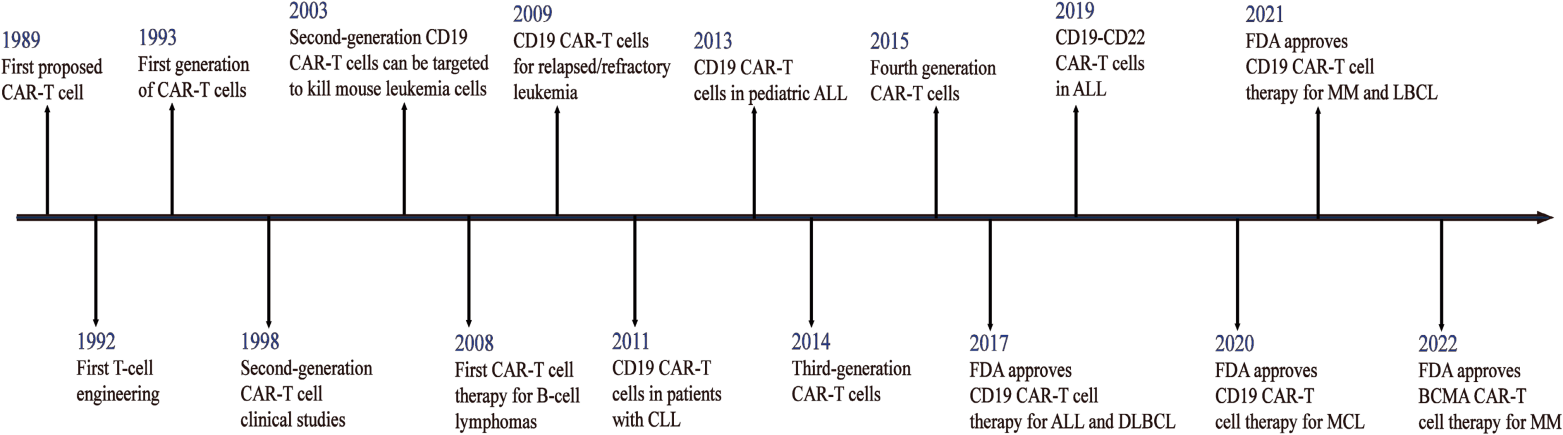
The history of CAR‐T cell's progress and milestones. CLL, chronic lymphocytic leukaemia; ALL, acute lymphoblastic leukaemia; DLBCL, diffuse large B‐cell lymphoma; MCL, mantle cell lymphoma; MM, multiple myeloma; LBCL, diffuse large B‐cell lymphoma.

From 1989, when Cross G and three other scientists first introduced the concept of ‘CAR’ in human history,^23^ to 2022, when the FDA approved BCMA CAR‐T cells for the treatment of multiple myeloma,^35^ it has been more than 30 years since the development of CAR‐T immune cell therapy (Figure 3). CAR‐T immune cell therapy has helped some patients with haematologic cancers achieve remissions of up to 10 years,^36^ and future breakthroughs in the field of CAR‐T cells are sure to follow, with major trends in the field involving targets, disease treatment modalities and more.

## 5 DIFFERENT TARGETS OF CAR‐T FOR AML

**FIGURE 4:**
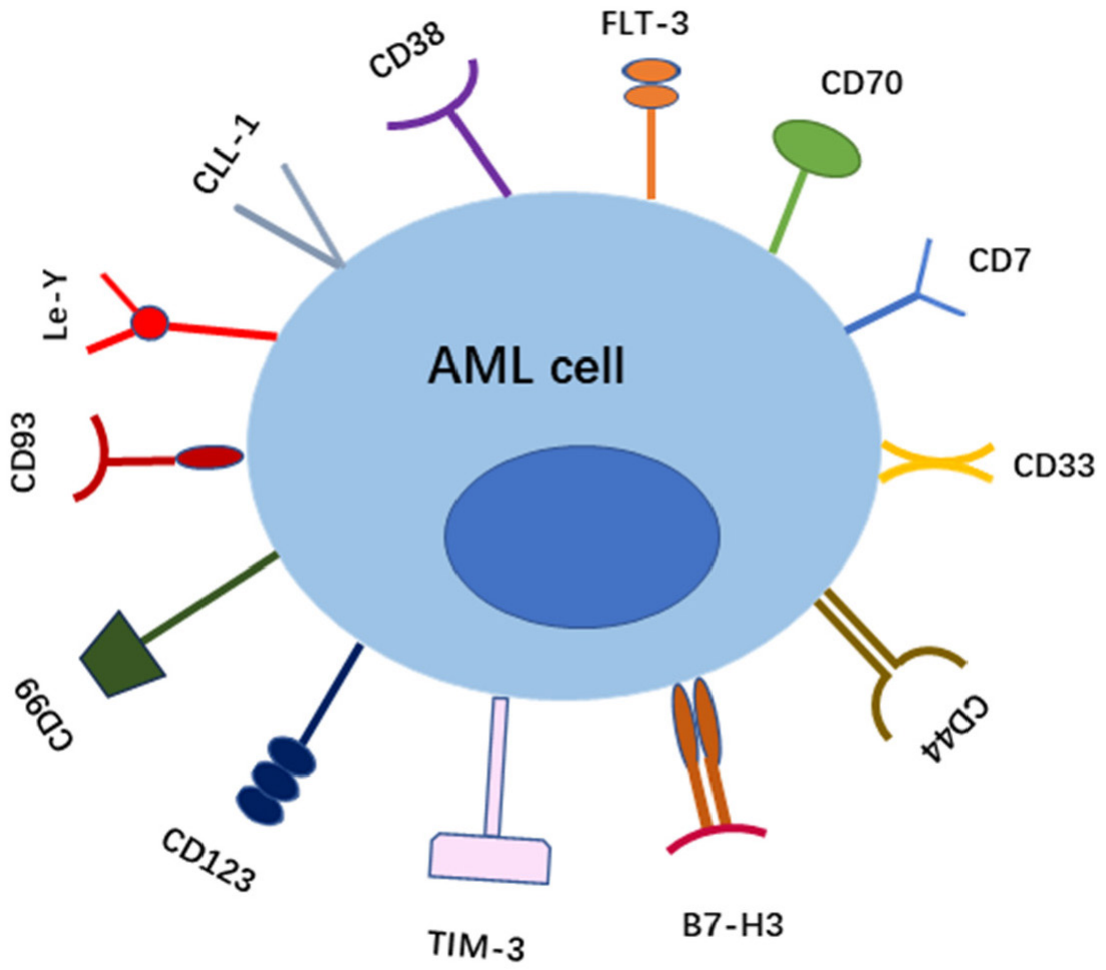
Diagram of different target antigens of AML.

In recent years, many studies have also screened and identified new target antigens for AML and designed various CAR‐T cells against these target antigens to evaluate their preclinical and clinical effects (Table 1). The latest advances in various targets are now discussed (Figure 4).

**TABLE 1:** Ongoing clinical trials of CAR‐T cells for acute myeloid leukaemia.

NCT number	Study title	Study status	Phases	Enrollment	Targets
NCT05445765	Anti‐CD33 CAR‐T Cells for the Treatment of Relapsed/Refractory CD33+ Acute Myeloid Leukaemia^131^	Not Yet Recruiting	1	10	CD33
NCT05248685	Optimized Dual CD33/CLL1 CAR‐T Cells in Subjects with Refractory or Relapsed Acute Myeloid Leukaemia^132^	Recruiting	1	20	CD33, CLL1
NCT03896854	CAR‐T Cell in CD19 Positive Relapsed or Refractory Acute Myeloid Leukaemia (AML)^133^	Recruiting	1,2	15	CD19
NCT05239689	Clinical Study of CD38 CAR‐T Cells in the Treatment of Hematological Malignancies^134^	Recruiting	Early 1	36	CD38
NCT01716364	Safety Study of Anti Lewis Y Chimeric Antigen Receptor in Myeloma, Acute Myeloid Leukaemia or Myelodysplastic Syndrome^135^	Unknown	1	6	Lewis Y
NCT05252572	Clinical Study of CLL1 CAR‐T cells in the Treatment of Hematological Malignancies^136^	Recruiting	Early 1	36	CLL1
NCT05473221	Evaluate the Safety and Efficacy of CD33 CAR‐T in Patients With R/R AML^137^	Not Yet Recruiting	1	20	CD33
NCT03126864	Study of Adoptive Cellular Therapy Using Autologous T Cells Transduced with Lentivirus to Express a CD33 Specific Chimeric Antigen Receptor in Patients with Relapsed or Refractory CD33‐Positive Acute Myeloid Leukaemia^138^	Terminated	1	11	CD33
NCT04803929	Clinical Study of Anti‐ILT3 CAR‐T Therapy for R/R AML(M4/M5)^139^	Recruiting	Early 1	25	ILT3
NCT03927261	PRGN‐3006 Adoptive Cellular Therapy for CD33‐Positive Relapsed or Refractory AML, MRD Positive AML or Higher Risk MDS^140^	Recruiting	1	88	CD33
NCT04318678	CD123‐Directed Autologous T‐Cell Therapy for Acute Myelogenous Leukaemia (CATCHAML)^141^	Recruiting	1	32	CD123
NCT05488132	Administration of Anti‐siglec‐6 CAR‐T Cell Therapy in Relapsed and Refractory Acute Myeloid Leukaemia (rr/AML)^142^	Recruiting	1,2	20	Siglec‐6
NCT04662294	CD 70 CAR‐T for Patients with CD70 Positive Malignant Hematologic Diseases^143^	Recruiting	Early 1	108	CD70
NCT04272125	Safety and Efficacy of CD123‐Targeted CAR‐T Therapy for Relapsed/Refractory Acute Myeloid Leukaemia^144^	Recruiting	1,2	40	CD123
NCT05949125	Dose‐escalating Trial with Allo‐RevCAR01‐T Cells in Combination with CD123 Target Module (R‐TM123) for Participants with Selected Hematologic Malignancies Positive for CD123^145^	Not Yet Recruiting	1	37	CD123
NCT02203825	Safety Study of Chimeric Antigen Receptor Modified T‐cells Targeting NKG2D‐Ligands^146^	Completed	1	12	NKG2D
NCT04835519	Phase I/II Study of Enhanced CD33 CAR‐T Cells in Subjects with Relapsed or Refractory Acute Myeloid Leukaemia^147^	Recruiting	1,2	25	CD33
NCT05454241	CD7 CAR‐T for Patients With r/r CD7+ Hematologic Malignancies^148^	Recruiting	1	22	CD7
NCT05945849	CD33KO‐HSPC Infusion Followed by CART‐33 Infusion(s) for Refractory/Relapsed AML^149^	Not Yet Recruiting	1	16	CD33
NCT05105152	PLAT‐08: A Study Of SC‐DARIC33 CAR‐T Cells in Paediatric and Young Adults with Relapsed or Refractory CD33+ AML^150^	Recruiting	1	18	CD33
NCT05467254	Evaluate the Safety and Efficacy of CLL1 + CD33 CAR‐T in Patients With R/R AML^151^	Not Yet Recruiting	1	20	CD33
NCT04257175	CAR‐T CD19 for Acute Myelogenous Leukaemia With t 8:21 and CD19 Expression^152^	Recruiting	2,3	10	CD19
NCT05943314	Clinical Study on Safety and Efficacy of Anti‐CLL1 /+CD33 CAR‐T Cells in the Treatment of Acute Myeloid Leukaemia^153^	Not Yet Recruiting	NA	5	CLL1 + CD33
NCT04599543	IL3 CAR‐T Cell Therapy for Patients with CD123 Positive Relapsed and/or Refractory Acute Myeloid Leukaemia^154^	Not Yet Recruiting	Early 1	36	CD123
NCT05023707	Anti‐FLT3 CAR‐T Cell Therapy in FLT3 Positive Relapsed/Refractory Acute Myeloid Leukaemia^155^	Recruiting	1,2	5	FLT3
NCT04351022	CD38‐targeted Chimeric Antigen Receptor T Cell (CAR‐T) in Relapsed or Refractory Acute Myeloid Leukaemia^156^	Recruiting	1,2	20	CD38
NCT05672147	CD33 CAR‐T Cell Therapy for the Treatment of Recurrent or Refractory Acute Myeloid Leukaemia^157^	Recruiting	1	27	CD33
NCT04219163	Chimeric Antigen Receptor T‐cells for The Treatment of AML Expressing CLL‐1 Antigen^158^	Recruiting	1	18	CLL1
NCT02623582	CD123 Redirected Autologous T Cells for AML^159^	Terminated	Early 1	7	CD123
NCT05266950	Safety and Efficacy Study of CI‐135 CAR‐T Cells in Subjects with Relapsed or Refractory Acute Myeloid Leukaemia^160^	Recruiting	1	7	CI‐135
NCT03672851	Study Evaluating Safety and Efficacy of CAR‐T Cells Targeting CD123 in Patients with Acute Leukaemia^160^	Terminated	1	2	CD123
NCT04762485	Humanized CD7 CAR‐T Cell Therapy for r/r CD7+ Acute Leukaemia^160^	Recruiting	1,2	20	CD7
NCT05377827	Dose‐Escalation and Dose‐Expansion Study to Evaluate the Safety and Tolerability of Anti‐CD7 Allogeneic CAR‐T Cells (WU‐CART‐007) in Patients with CD7+ Hematologic Malignancies^160^	Not Yet Recruiting	1	48	CD7
NCT03795779	CLL1‐CD33 cCAR in Patients with Relapsed and/or Refractory, High Risk Hematologic Malignancies^160^	Unknown	Early 1	20	CLL1, CD33
NCT05654779	CLL‐1/CD33 Targeted LCAR‐AMDR Cells in Patients with Relapsed or Refractory Acute Myeloid Leukaemia^160^	Recruiting	1	34	CLL1/CD33
NCT04265963	CD123‐Targeted CAR‐T Cell Therapy for Relapsed/Refractory Acute Myeloid Leukaemia^160^	Recruiting	1,2	45	CD123
NCT05016063	Dual CD33‐CLL1‐CAR‐T Cells in the Treatment of Relapsed/Refractory Acute Myeloid Leukaemia^160^	Not Yet Recruiting	Early 1	32	CD33
NCT03556982	CAR‐T‐123 for Relapsed/ Refractory Acute Myelocytic Leukaemia(AML)^160^	Unknown	1,2	10	CD123
NCT03585517	Safety and Efficacy Evaluation of IM23 CAR‐T Cells (IM23CAR‐T)^160^	Completed	1	10	IM23
NCT04658004	NKG2D CAR‐T Cell Therapy for Patients with Relapsed and/or Refractory Acute Myeloid Leukaemia^160^	Not Yet Recruiting	Early 1	36	NKG2D
NCT05442580	CAR‐T‐38 in Adult AML and MM Patients^160^	Recruiting	1	36	CD38
NCT03766126	Lentivirally Redirected CD123 Autologous T Cells in AML^160^	Active, not recruiting	1	12	CD123
NCT03796390	Study Evaluating Safety and Efficacy of CAR‐T Cells Targeting CD123 in Patients with Acute Myelocytic Leukaemia^160^	Unknown	1	15	CD123
NCT04156256	CD123‐CD33 cCAR in Patients with Relapsed and/or Refractory, High Risk Hematologic Malignancies^160^	Unknown	Early 1	20	CD123‐CD33
NCT04678336	CD123 Redirected T Cells for AML in Paediatric Subjects^160^	Active, not recruiting	1	12	CD123
NCT05445011	Anti‐FLT3 CAR‐T Cell (TAA05 Cell Injection) in the Treatment of Relapsed / Refractory Acute Myeloid Leukaemia^160^	Recruiting	1	12	FLT3
NCT04014881	Safety and Efficacy of Anti‐CD123 CAR‐T Therapy in Patients with Refractory/ Relapsed CD123+ Acute Myeloid Leukaemia^160^	Unknown	1	50	CD123
NCT02799680	Allogeneic CAR‐T‐33 for Relapsed/Refractory CD33+ AML^161^	Unknown	1	12	CD33
NCT04033302	Multi‐CAR‐T Cell Therapy Targeting CD7‐positive Malignancies^161^	Recruiting	1,2	30	CD7
NCT05432401	TAA05 Injection in the Treatment of Adult Patients with FLT3‐positive Relapsed/Refractory Acute Myeloid Leukaemia^161^	Recruiting	Early 1	18	FLT3
NCT05463640	Evaluate the Safety and Efficacy of ADGRE2 CAR‐T in Patients With R/R AML^161^	Not Yet Recruiting	1	20	ADGRE2
NCT03904069	Study Evaluating the Safety, Tolerability, and Efficacy of FLT3 CAR‐T AMG 553 in FLT3‐positive Relapsed/Refractory AML^161^	Withdrawn	1	0	FLT3
NCT03631576	CD123/CLL1 CAR‐T Cells for R/R AML (STPHI_0001)^161^	Unknown	2,3	20	CD123/CLL1
NCT05467202	Evaluate the Safety and Efficacy of CLL1 CAR‐T in Patients With R/R AML^161^	Not Yet Recruiting	1	20	CLL1
NCT01864902	Treatment of Relapsed and/or Chemotherapy Refractory CD33 Positive Acute Myeloid Leukaemia by CAR‐T‐33^161^	Unknown	1,2	10	CD33
NCT04884984	Anti‐CLL1 CAR‐T Cell Therapy in CLL1 Positive Relapsed/Refractory Acute Myeloid Leukaemia (AML)^161^	Recruiting	1,2	20	CLL1
NCT03114670	Donor‐derived Anti‐CD123 CAR‐T Cells for Recurred AML After Allo‐HSCT^161^	Unknown	1	20	CD123
NCT03971799	Study of Anti‐CD33 Chimeric Antigen Receptor‐Expressing T Cells (CD33 CAR‐T) in Children and Young Adults with Relapsed/Refractory Acute Myeloid Leukaemia^161^	Recruiting	1,2	37	CD33

*Note*: CAR‐T cell therapy for AML on Clinicaltrials.gov as of 7 August 2023.

### 5.1 CD33

CD33, also known as SIGLEC3, is a single‐pass type I membrane protein that belongs to the immunoglobulin superfamily and SIGLEC (sialic acid binding Ig‐like lectin) family.^37^ Studies have demonstrated that CD33, a myeloid differentiation antigen, is highly expressed in acute myeloid leukaemia progenitor cells,^38^ but it also can be detected in normal haematopoietic stem cells.^39^ Thus, it was used as a target for antibody‐based cancer therapy for years. Gemtuzumab ozogamicin (GO), the first antibody‐drug conjugate (ADC) for AML treatment, was approved by the FDA in 2017.^40^ When used with other traditional drugs, the long‐term survival rate can reach over 70%.^39^ The potential for long‐lasting marrow clearance by CD33 CAR‐T cells has led to deep concerns, and numerous methods are being investigated to reduce the side effects. Through blood cell restoration, bispecific CAR‐T or genetic modification of CD33 CAR‐T vector, CD33 CAR‐T clinical trials are currently undergoing.^37^, ^41^, ^42^

### 5.2 CD123

CD123, the interleukin 3 (IL‐3) receptor alpha chain, is structurally a type I membrane protein with three extracellular areas, a transmembrane region and an intracellular portion.^43^ CD123 was considered a potential target, highly expressed on leukaemia stem cells (LSCs) and acute myeloid leukaemia (AML) primordial cells like other AML markers.^44^ CAR‐engineered T cells that target CD123 can cause myeloids to be cleared from immunodeficient mouse models of human AML reference. In vivo, co‐stimulated 4‐1BB CAR‐T cells were able to eliminate transplanted primary human AML cells, but a xenograft model revealed impaired haematopoiesis.^45^ Research has revealed that 70% of AML cells express CD33 and CD123, which supports the case for targeting both of these molecules simultaneously in AML treatment.^46^ Bispecific CARs with CD33 and CD123 single‐stranded variable fragments (ScFv) have superior proliferative capability and less HSC toxicity both in vitro and in vivo.^47^ Many clinical studies including numerous antigens have been started based on preclinical data.^48^, ^49^, ^50^ Yet, a significant issue with the development of AML CAR‐T treatments is the probable loss of proper bone marrow and haematopoietic precursor cells.^51^, ^52^ These antigens were selected because studies have consistently demonstrated that they express more strongly on AML cells than on healthy haematopoietic stem cells (HSCs), indicating that they have little effect on healthy haematopoiesis.

### 5.3 CLL‐1

CLL‐1, also known as CLEC12A, is a type II transmembrane glycoprotein that belongs to clade V of the C‐type lectin‐like receptor family.^53^ According to research, the glycoprotein C‐type lectin‐like molecule‐1 (CLL‐1) expressed in myeloid cells, AML blasts and leukaemia stem cells, while hard to detect in haematopoietic stem cells.^54^ AML cell lines and transgenic mice models have been used to test the efficiency of many CLL‐1 CAR‐T cells. Due to the powerful in vitro effects and eradication of leukaemic cells in mice models without significant myelotoxicity, CLL‐1 is theoretically a promising target for AML. Notably, CLL‐1 was also expressed in some early haematopoietic cells and normal monocytes, indicating the potential off‐target risk.^55^ Both CLL‐1+ progenitor cells and mature granulocytes were effectively eliminated, albeit to varied degrees, after co‐culturing normal autologous HSCs with CLL‐1 CAR‐T cells from healthy normal donors.^56^ Targeting CLL‐1 has significant drawbacks due to the antigen's differential expression in mature myeloid cells. The caspase‐9 suicide gene system was thus developed to control CLL‐1 CAR‐T cells and reduce the damage to normal myeloid cells.^57^

### 5.4 CD70

CD70, a type 2 transmembrane glycoprotein from the tumour necrosis factor (TNF) family, is a compromising target for CAR‐T cell therapy in AML because it is present on both leukaemic blasts and leukaemia stem cells in AML patients. But different from CD33 or CD123, CD70 barely can be detected in normal HSCs.^58^ In tumour cell lines and animal xenograft models, it was discovered that CD27z‐CARs, which comprise CD70 ligand and CD3z chain fusion, displayed superior proliferative and anticancer activity than CD70 scFv CARs while retaining normal HSCs.^59^ The Marcela Maus laboratory evaluated the anti‐tumor activity of CD70 CAR‐T cells through the NSG Molm13 AML model and found that in co‐culture with AML, CD70‐CAR‐T cell activity was impaired by the soluble CD27 form was attenuated.^60^ In response to the soluble ligand limitation, a series of new hinge CAR variants (truncated, deletion, flexible and ‘CD8 hinge & TM’) were evaluated, and the CD8 hinge and TM variants were found to have enhanced anti‐tumour activity and better expansion capacity in vitro and in vivo compared to natural 41BB‐based CAR‐T cells. A clinical trial using CD70 CAR‐T cells is currently being conducted for the therapy of individuals with CD70‐positive haematologic malignancies due to minimal myelotoxicity (NCT04662294).

### 5.5 CD38

CD38 is a type II transmembrane glycoprotein, one of the target antigens of AML, which is expressed in AML progenitor cells but not in normal human haematopoietic stem cells.^61^ It is expressed in a variety of immune cells, including B‐lymphocytes, T‐lymphocytes, monocytes and NK cells, and influences tumour‐killing activity by regulating the release of cytokines.^62^ The number and intensity of CD38 antigens, which are expressed by over 80% of AML cells, should be increased to achieve better results when designing CD38‐CAR‐T cells against these cells. Accordingly, CD38 expression in AML cells can be induced by all‐trans retinoic acid (ATRA), a treatment agent for acute promyelocytic leukaemia (APL). In preclinical research, all‐trans retinoic acid (ATRA) and CD38 CAR‐T cells were coupled to treat AML cell lines, and increased CD38 expression strengthened the cytotoxic effect on AML cell lines.^63^, ^64^ If ATRA is not available, the cytotoxic effect of CD38 CAR‐T cells is limited.^64^ In another study, CD38 CAR‐T‐cell therapy was administered to six patients with AML who relapsed after Allo‐HSCT,^65^ and the study showed that CR was achieved in four cases, grades 2–3 CRS were seen in 88% of cases, and grades III and IV haematologic toxicity was seen in all three patients.

### 5.6 Le Y

Lewis Y (Le Y) is a carbohydrate tumour‐associated antigen that promotes tumour survival, invasion and metastasis.^66^ Le Y is abundantly expressed in haematologic tumours, including acute myeloid leukaemia, and is minimally expressed in healthy tissues, making Le Y a potentially suitable target for the treatment of AML.^67^ In a preclinical study, investigators tested the effectiveness of anti‐Le Y CAR‐T cells against AML and MM (multiple myeloma) cells.^67^ The Peter MacCallum Cancer Center is conducting an important clinical study evaluating the potential of CAR‐T therapy in patients with AML, with a phase I clinical trial demonstrating the feasibility and safety of targeting Lewis Y and CAR‐T cells in skin and bone marrow in proven leukaemia for up to 10 months.^68^ It has been reported that Le Y CAR‐T cells specifically target Le Y+ cells by producing interferon‐γ. Neeson et al. designed anti‐Lewis Y CAR‐T cells using autologous T cells and found that high levels of interferon‐γ were produced in the killing of Lewis Y‐positive AML cells.^69^

### 5.7 CD7

Transmembrane glycoprotein CD7, which is produced by T cells, NK cells and cord blood myeloid progenitor cells, has a synergistic stimulatory effect on the contacts between B and T cells during lymphatic development. AML cells express CD7, while healthy myeloid cells do not, and CD7 is not harmful to normal cells.^70^ Consequently, it might be a suitable target for the elimination of malignant cells. since T cells also express CD7, removal of the CD7 gene in T cells is critical for successful treatment. In two experiments, Gomes‐Silva et al. used a transgenic model of AML and CD7 CAR‐T cells to target CD7+ tumour cells.^71^ The CD7 gene was deleted from primary activated T cells using a CRISPR/Cas9 technique before producing CAR‐T cells. Then, by employing scFv generated from anti‐CD7 antibodies, second‐generation CD28‐ CD3‐CD7 CAR ‐T cells were developed with de novo CD7 knockout. Surprisingly, the study's findings revealed that their CAR‐T cells had a strong killing impact on AML, significantly eliminating both primary AML cells and leukaemic colony‐forming cells while causing no harm to healthy cells or erythrocytes. Therefore, CD7 CAR‐T cells may be a potential successful therapy for refractory or relapsed AML.

### 5.8 FLT‐3

FMS‐like tyrosine kinase‐3 (Flt‐3) is a glycosylated protein of the class III receptor tyrosine kinase family,^72^ which is mainly expressed on haematopoietic stem cells and myeloid cells and participates in normal haematopoiesis by controlling cell survival, proliferation and differentiation.^73^ Flt3 expression is increased on primitive cells in 70% of AML patients, and 30% of AML patients are associated with mutations in Flt3.^74^ Midostaurin played a favourable role in suppressing AML and improving survival when combined with chemotherapeutic agents.^75^ In a preclinical study, investigators evaluated the killing effect of second‐generation Flt3‐41BB CAR‐T cells in a xenograft mouse model and found that the mice prolonged their lives after input of Flt3‐41BB CAR‐T cells.^76^ In another preclinical study, second‐generation CAR‐T cells with Flt3 ligands prolonged the survival of Flt3 mutant mice, showing limited myelotoxicity.^64^, ^77^

### 5.9 B7‐H3

B7‐H3, also known as CD273, is a type I transmembrane protein expressed in a variety of cancer types including AML mother cells.^78^ As a T‐lymphocyte stimulating protein, B7‐H3 reduces T‐cell cytotoxicity and promotes immune evasion within cancer cells.^79^ Lichtman et al. evaluated the ability of B7‐H3‐specific CAR‐T cells to combat leukaemia in an in vitro xenograft model, which was found to be cytotoxic and show significant interferon‐γ and IL‐2 release in AML cell lines at an E: T ratio of 1:5.^80^ Yang et al. designed tandem anti‐B7‐H3 and anti‐CD70 CAR‐T cells, and both in vitro and in vivo experiments showed significant antigenic toxicity but little damage to haematopoietic cells.^81^ More research is needed on B7‐H3 as a potential CAR‐T cell target for the treatment of AML.

### 5.10 TIM‐3

T‐cell immunoglobulin mucin‐3 (Tim‐3) is a transmembrane protein belonging to the TIM family that is expressed on almost all forms of AML primitive cells but not on HSCs, making it a promising target.^82^ The main cause of leukaemia relapse after chemotherapy is resistance to LSCs because they cannot target the elimination of LSCs. In one study, researchers created a phage library from which anti‐human TIM antibodies were isolated and evaluated the killing ability of second‐generation TIM3 CAR‐T cells against leukaemia cell lines and primary AML cells in vitro and found that they had good anti‐leukaemia activity.^83^ In another study, Xin He et al. evaluated the therapeutic efficacy of TIM‐3 CAR‐T cells in vivo through a xenograft model and found that the levels of cytotoxic agents, such as interferon‐gamma, granzyme B, perforin, and interleukin 5, were markedly increased, and that the TIM‐3 CAR‐T cells were effective in killing LSCs.^84^ Effective CAR‐T therapies are being developed one after another, and some studies have shown that CD13 and TIM3 bispecific CAR‐T cells have a better killing effect on AML, and also reduce the cytotoxicity of stem cells and peripheral myeloid cells.^84^

### 5.11 CD44v6

CD44v6 is a splice variant of the hyaluronan CD44 receptor and belongs to class I membrane glycoproteins. It is highly expressed in other haematologic malignancies, including acute myeloid leukaemia, and is not expressed on healthy progenitor cells, and haematopoietic stem cells^85^, ^86^; therefore, CD44v6 is expected to be an effective target antigen for AML therapy. Casucci et al. evaluated the killing ability of second‐generation CD44v6 CAR‐T cells targeting CD44+ AML cells using scFv with humanized anti‐CD44v6 single‐chain antibody. The results showed that CD44v6 CAR ‐T cells produced anti‐tumour cytokines and effectively cleared CD44+ tumour cells.^86^ Notably, CD44v6 CAR‐T cells produced haematological toxicity, so‐called extra‐tumour toxicity, to monocytes during the selective removal of tumour cells. To avoid this side effect, non‐immune inducible Caspase9 (IC9) and thymidine kinase suicide genes were used to efficiently eliminate CAR‐T cells on CD44v6 CAR‐T cells.^87^ DNA methylation has been associated with CD44v6 expression in AML cells and T‐cell exhaustion and function, and in AML therapy, the hypomethylating agents (HAMs) decitabine (Dec) and azacitidine (Aza) have been widely used.^88^, ^89^ A recent study has shown that combining Dec or Aza with CD44v6 CAR‐T cells improves the function of CD44v6 CAR‐T cells and enhances anti‐tumour capacity against AML.^90^

### 5.12 CD93

CD93, also known as C1QR1, is a cell surface C‐type lectin transmembrane receptor protein that is differentially expressed in approximately 55% of AML patients, but not in HSC or other precursor myeloid cells.^91^ CD93 plays an important role in host defence and intercellular adhesion. Recently, Richards et al. developed a new humanized CD93‐specific binding protein and evaluated the role of CD93 CAR‐T cells in leukaemia, and in vivo experiments showed that CD93 CAR‐T cells specifically cleared AML but not HSPC.^92^ Notably, human endothelial cells express CD93, so CD93 CAR‐T cells exert a targeted, non‐tumorigenic toxic effect on vascular endothelial cells. To limit the cytotoxic effects of CAR‐T cell recognition and killing, Richards et al. recently developed a non‐gated anti‐CD93 CAR‐T cell.^92^

### 5.13 CD99

CD99 is a transmembrane glycoprotein and is strongly expressed in the majority of AML. CD99 is a marker expressed on the surface of leukaemic stem cells but not normal haematopoietic stem cells, thus can be a promising therapeutic target for AML.^16^, ^93^ Previous studies show that expression of CD99 can separate LSCs from functionally normal HSCs in AML and have higher CD99 levels in relapse AML blasts.^94^ CD99 single‐chain variable fragment antibody was proved to induce apoptotic cell death in AML cell lines and extended the survival time of AML xenograft mouse model.^95^ Currently, purine nucleotide analogs clofarabine and cladribine that can inhibit CD99 dimerization were approved by the FDA to treat Ewing's sarcoma.^96^, ^97^ CD99 drugs for AML are still under development.

## 6 EXPRESSION OF AML TARGETS

**FIGURE 5:**
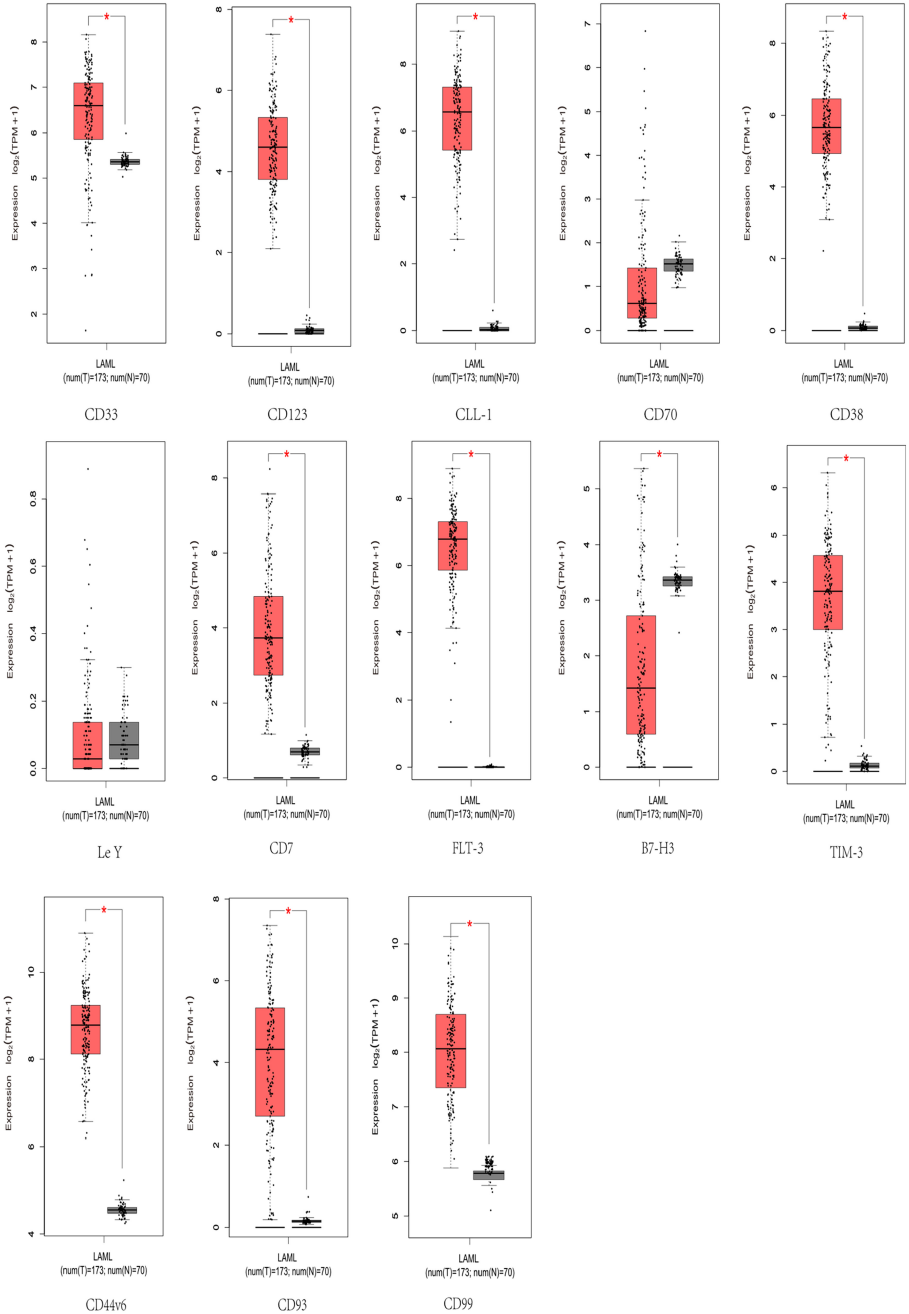
Expression analysis of AML targets in the GEPIA database. The filter conditions are *p*‐value <0.05 and the absolute value of the difference (| log2 (Fold Change) |) > 1.

Annotation of antigen expression can predict potential targeted tumour effects. We performed expression analysis of the above AML targets based on the Gene Expression Profiling Interactive Analysis (GEPIA) database in the hope of helping researchers select appropriate antigens. The results showed that the expression of CD33, CD123, CLL‐1, CD38, CD7, FLT‐3, B7‐H3, TIM‐3, CD44V6, CD93 and CD99 was significantly higher than that of normal tissues in tumour tissues, and the expression of CD70 and Le Y in tumour tissues was not significantly different from that of normal tissues (Figure 5). Ideal targets should ensure that CAR‐T cells specifically kill tumour cells while not harming or rarely harming normal cells, which is difficult to achieve in practice. Analysing the expression of various potential targets can provide researchers with some ideas, such as using two single‐targeted CAR‐T cells in combination, dual‐targeted CAR‐T cells, triple‐targeted CAR‐T cells and tandem CAR‐T cells, to overcome off‐target effects, antigen escape, and other problems.

## 7 LIMITATIONS AND CHALLENGES OF CAR‐T FOR AML

**FIGURE 6:**
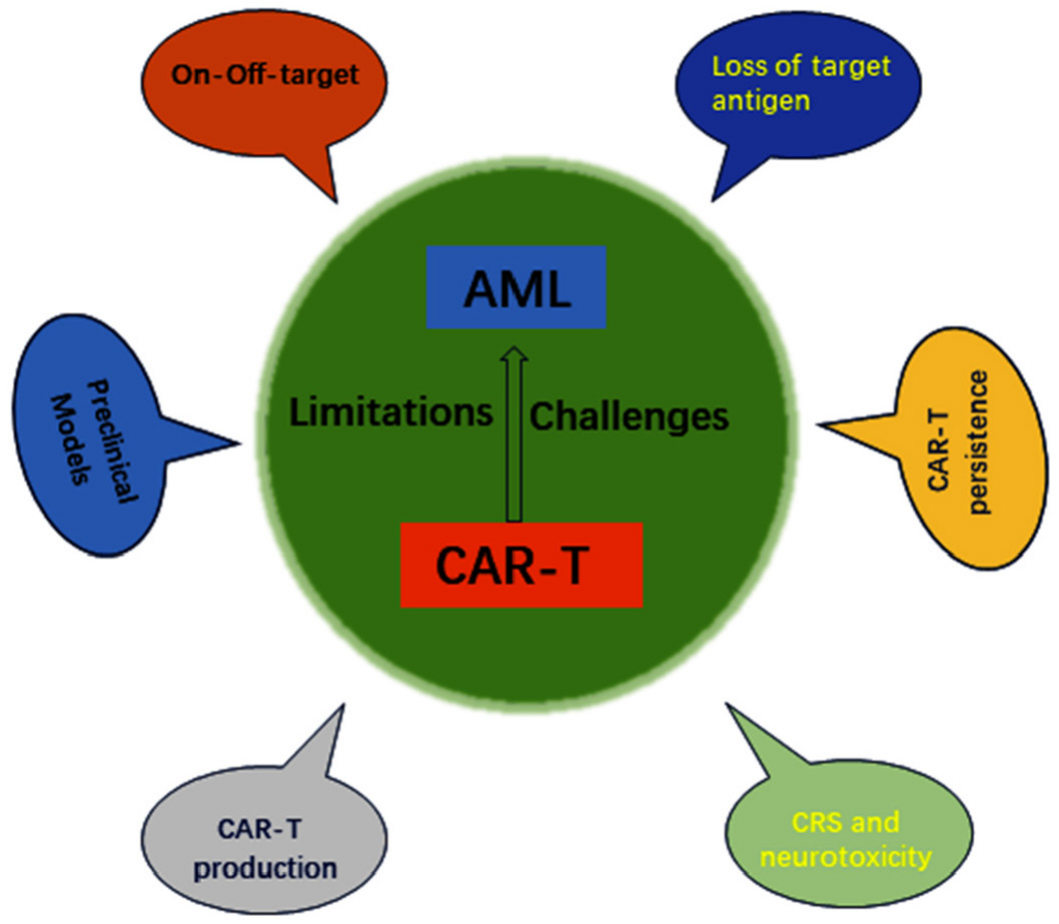
Limitations and challenges of CAR‐T cell therapy for acute myeloid leukaemia.

Despite the tremendous development of CAR‐T cells in the treatment of haematologic malignancies, however, CAR‐T cells still face many problems in the treatment of AML, such as target antigen loss, off‐target effects, cytokine release syndrome (CRS) and neurotoxicity, CAR‐T persistence, preclinical models and CAR‐T production. We summarize the current dilemmas facing CAR‐T cell therapy for AML (Figure 6).

### 7.1 Antigen loss

The toxic effect of CAR‐T cells on tumours is dependent on the recognition and binding of CAR antigens, and loss or down‐regulation of target antigens results in AML progenitor cells evading immune surveillance, thus limiting the killing effect of CAR‐T cells.^98^ Despite the CAR‐T cell therapy's fast advancement, treatment resistance has grown to be a significant barrier to its use in clinical settings. The cause of most resistance in CAR‐T cell therapy is target antigen loss. The CAR study CTL109 by Novartis (NCT01626495) demonstrated an amazing 93% efficiency.^99^ Yet, the likelihood of a complete response after 1 year has dropped to about 55%, indicating that drug resistance developed in about 50% of patients during this period. This implies that any CAR‐T cell therapy may not be effective enough if it just targets one antigen.

### 7.2 On–Off‐target effects

On–off‐target effects include normal cell on‐target and tumour cell off‐target. Normal cell on target means that normal cells expressing target antigens are inevitably killed by CAR‐T, whereas tumour cell off‐target means that the density of target antigens expressed by tumour cells is reduced or lost, resulting in the inability of CAR‐T to recognize and attack tumour cells. For example, CD19‐CAR‐T cells recognize and kill B‐cell lymphoma and leukaemia cells while also killing normal CD19‐positive B‐cells in the patient. Due to the target antigens' high levels of expression on normal HSC, off‐target consequences such as haematological toxicity and protracted myeloid elimination are some of the main drawbacks of CAR‐T cells in the therapy of AML.^100^ B‐cell malignancies' CAR‐T cells specifically target CD19 and their depletion can be managed without endangering life. Acute myeloid leukaemia lacks a disposable antigen, unlike B‐cell malignancies, and targeting myeloid antigens causes long‐term myelotoxicity. Prolonged myelotoxicity raises the risk of sepsis as well as invasive fungal infections, which is clinically undesirable.

### 7.3 CRS and neurotoxicity

Systemic immune inflammation in which patients rapidly produce and secrete inflammatory cytokines after injection of CAR‐T cells is called CRS. CRS is one of the major side effects of CAR‐T cell therapy because when CAR‐T cells exert their effector functions, large amounts of cytokines, such as IL‐6, IFN‐γ, IL‐1β, GM‐CSF and TNF‐α production, cause a systemic inflammatory response, leading to signs such as hypoxia, fever, myalgia, hypotension and neurologic dysfunction, which threaten patients' lives.^101^, ^102^ The main mechanism by which CRS occurs is the disruption of the balance between pro‐ and anti‐inflammatory in the body, where monocytes are activated and secrete cytokines such as IL‐6 and IL‐1 in response to the interaction of CAR‐T cells and tumour cells, followed by peripheral molecules that initiate the activation of endothelial cells and initiate pro‐inflammatory pathways, thus disrupting the balance between pro‐ and anti‐inflammatory.^103^ Neurotoxicity generally occurs a few days after the onset of CRS, when excessive cytokines diffuse in the brain, and patients may experience adverse conditions, such as mild headaches, hallucinations, confusion, psychosis and seizures.^104^, ^105^

### 7.4 CAR‐T persistence

Clinical trials have confirmed that reduced CAR‐T cell persistence is one of the major reasons for CAR‐T cell therapy failure. CAR‐T cells do not sustain their tumour‐killing effect in patients due to immunorecognition of CAR‐derived exogenous peptides and subsequent disruption of immune‐mediated modification of T‐lymphocytes.^106^, ^107^ CAR‐T cells in patients must be used for a longer period to destroy AML cells. According to data from the original CD19 CAR in the juvenile B‐cell ALL research, the ideal length of CAR‐T cells to prolong remission is up to 8–10 months, which seems unlikely in AML patients given the danger of infection and its possibly fatal consequences.

### 7.5 Preclinical models

Fundamental in vitro processes including proliferation, antigen‐targeted lysis, cytokine production and allergen stimulation tests are examples of quantitative processes that may not be able to properly predict in vivo performance. Utilizing preclinical animal models of adaptive T cell transfer for transplantation, the CAR‐T cell design was validated. The immunological microenvironments in mice and human lymphoid organs are so different from one another, however, that these mouse models do not always adequately describe human T cell function, despite their initial effectiveness in removing tumour cells.^108^

### 7.6 CAR‐T production

Unlike other immunotherapeutic methods, CAR‐T cell therapy is highly personalized, which requires the isolation of T cells from the patient and their modification and transformation in vitro by genetic engineering before being infused back into the patient's body. CAR‐T cells cannot be mass‐produced or generalized, and the constraints in the whole process of CAR‐T production and treatment include cost, raw materials, equipment, environment, etc. CAR‐T cells can be costly due to the complex preparation process. Culture media and reagents used to better enable cell expansion are not yet standardized, and raw materials and reagents may be difficult to obtain if manufactured according to GMP specifications. A variety of advanced instruments and techniques are required to genetically modify T cells. In addition, a highly sterile and controlled environment and continuous monitoring are essential for patients undergoing CAR‐T cell therapy. These constraints are undoubtedly challenging for CAR‐T cell therapy for haematologic tumours.^109^, ^110^

## 8 OPTIMIZATION STRATEGIES RELATED TO CAR‐T FOR AML

**FIGURE 7:**
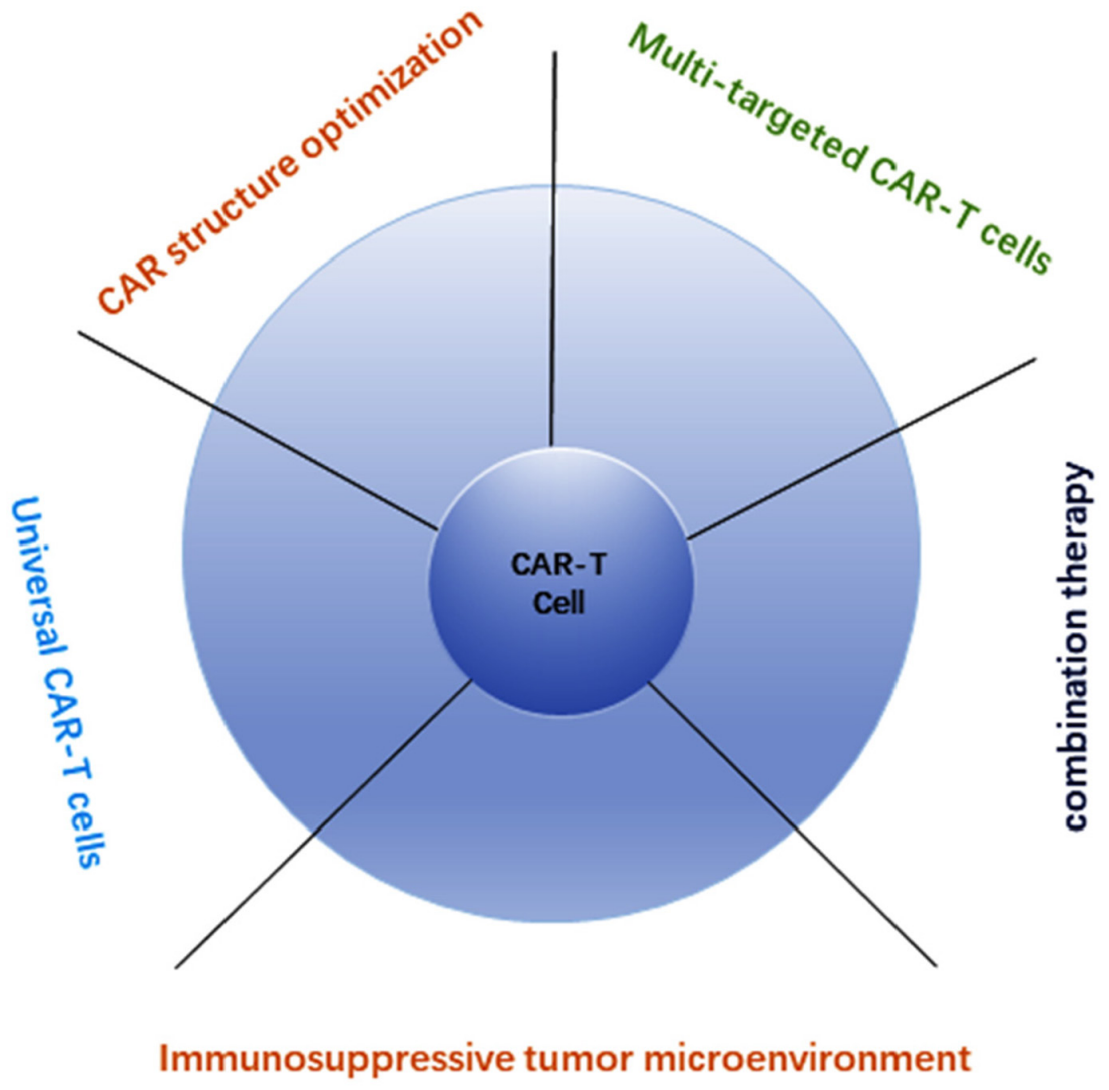
Schematic diagram of optimization strategy for CAR‐T cell therapy for AML.

To address the many problems faced in CAR‐T cell therapy for AML, several studies have been undertaken to improve the efficacy of CAR‐T cells. Such as CAR structure optimization, multi‐targeted CAR‐T cells, universal CAR‐T, combination therapy and immunosuppression of tumour microenvironment. We summarize current optimization strategies related to enhancing CAR‐T efficacy (Figure 7).

### 8.1 CAR structure optimization

Two important features of successful CAR‐T cell therapy are durability and efficacy, and factors such as weak CARS structure, lack of co‐stimulatory molecules, and insufficient expansion or activation in vitro and in vivo can affect the durability and efficacy of CAR‐T. CD28 or 4‐1BB stimulation of structural domains can enhance the durability of CAR‐T cells and their function.^111^, ^112^ The third‐generation CAR showed a strong anti‐tumour response compared to the first‐ and second‐generation CARs.^113^ CD27 and CD28 are the two most important co‐stimulatory receptors for T‐cell differentiation, with CD28 co‐stimulatory signals mainly triggering cell proliferation and CD27 co‐stimulatory signals promoting cell survival and differentiation. CAR‐T cells using CD27 co‐stimulators are impressive in terms of safety. Another beneficial co‐stimulatory molecule, TLR2, has also been used to enhance the anti‐tumour activity of CAR‐T cells.^114^ Notably, CAR‐T cells with MyD88/CD40 structural domains showed potential killing effects on tumour cells.^115^ For the survival and effectiveness of CAR‐T cells to become better, powerful co‐stimulatory molecules arming CAR‐T cells or modifying optimized CAR‐T cells are necessary.

### 8.2 Multi‐targeted CAR‐T cells

CAR‐T cells targeting only one antigen cannot be used in all AML patients because tumour‐associated antigens (TAAs) on the surface of tumour cells are different in different pathologies, and the TAAs targeted by CAR‐T cells are also present in normal tissue cells, resulting in the risk of the inadvertent killing of their normal cells during the treatment process. In addition, the escape, reduction or loss of tumour antigens can directly reduce the effectiveness of CAR‐T cells. To overcome the problems of off‐target effect and antigen escape, a multi‐targeted CAR‐T cell strategy has been introduced into clinical trials.^33^, ^116^ Multi‐targeted CAR‐T cell applications include dual‐targeted CAR‐T cells, triple‐targeted CAR‐T cells, a combination of two single‐targeted CAR‐T cells, and tandem CAR‐T cells. Dual‐targeted and triple‐targeted CAR‐T cells refer to the design of two or three separate CARs on a single T‐lymphocyte, each targeting a different antigen, thus providing dual or triple targeting. The combination of two CAR‐T cells refers to two single‐targeted CAR‐T cells, each CAR targeting a separate antigen to improve tumour recognition. Tandem CAR‐T cells are two different scFv regions within one CAR, each targeting a different antigen. The researchers used Boolean logic operations to elucidate how these various types of multi‐targeted CAR‐T cells function, including ‘AND’, ‘OR’ and ‘NOT’. CAR‐T cells with ‘AND’ are activated only if both target antigens are recognized, CAR‐T cells with ‘OR’ need either of the two target antigens to be recognized to be activated, and CAR‐T cells with ‘NOT’ avoid killing normal cells by stopping when a specific antigen is detected.^117^ Therefore, the Boolean logic operations ‘AND’, ‘OR’ and ‘NOT’ in logic gates can improve the recognition and killing ability of CAR‐T cells on tumour cells and can also effectively avoid off‐target effects.

### 8.3 Universal CAR‐T cells

Based on the second‐generation CAR‐T, Cartellieri et al. designed a flexible CAR platform, the universal CAR‐T cell, utilizing CD28 as a co‐stimulatory structural domain.^118^ The universal CAR‐T cell has a switch structure consisting of two components that can be utilized to regulate the function of the CAR‐T cell. Notably, no extra‐tumour toxicity or xenograft‐versus‐host response was demonstrated in experiments using the universal CAR dual‐targeting CD33 and CD123 to lysate AML primary cells and AML cell lines. Graft‐versus‐host response (GVHR) or host‐versus‐graft response (HVGR) affects CAR‐T therapeutic efficacy, and the development of universal CAR‐T cell therapies necessarily addresses the immune rejection induced by allogeneic cell therapy. A commonly adopted means is to knock out the TCR of CAR‐T through gene editing technology to reduce its recognition and attack on patients and reduce the risk of GvHD. Knockdown of HLA‐like molecules responsible for antigen presentation reduces the immunogenicity of allogeneic‐derived CAR‐T cells and enhances their killing and expansion activities.^119^, ^120^

### 8.4 Combination therapy

Haematopoietic stem cell transplantation (HSCT), oncolytic viruses, checkpoint inhibitors and chemotherapeutic agents in combination with CAR‐T cells have shown great potential as several new therapeutic strategies in the treatment of human haematological malignancies.CD33 CAR‐T cells in combination with Allo‐HSCT have shown less haematopoietic toxicity in patients with CD33+ AML.CD33 CAR‐T cells eliminated CD33+ primitive cells without effect on CD33‐haematopoietic stem cells (HSPC).^121^, ^122^ The use of chemokine‐encoding gene vectors of lysosomal viruses allows for the recruitment of more CAR‐T cells, prolonging the duration of CAR‐T cells and directly attacking malignant cells, thereby increasing the durability and effectiveness of CAR‐T cells.^123^, ^124^ PD‐1, or programmed death receptor 1, is the most common immune checkpoint identified for T cells. PD‐1 regulates the immune system by down‐regulating the immune system's response to human cells and by suppressing the inflammatory activity of T cells. PD‐1 inhibitors have been the hottest method of tumour immunotherapy in recent years. By blocking the PD‐1/PD‐L1 signalling pathway with PD‐1 inhibitors, CAR‐T cells can target PD‐L1‐expressing tumour cells and play the role of tumour killing. In addition, the combination of corticosteroids and tocilizumab with CAR‐T cell therapy can reduce side effects in patients.^125^ Both drugs rapidly reverse CRS symptoms in most patients, but the long treatment interval between the two drugs Precise regulation of CAR‐T cell proliferation and differentiation is also needed to mitigate CRS. Siltuximab, an IL‐6 antagonist monoclonal antibody that prevents IL‐6 translocation from the blood–brain barrier (BBB), also plays an important role in managing neurotoxicity.^126^

### 8.5 Immunosuppressive tumour microenvironment

Tumour microenvironment (TME) refers to the surrounding microenvironment in which tumour cells exist, including surrounding blood vessels, immune cells, fibroblasts, bone marrow‐derived inflammatory cells, various signalling molecules and extracellular matrix (ECM). Depletion and decreased activity of CAR‐T cells are strongly associated with immunosuppression of TME.^101^, ^127^ Synergistic treatment with checkpoint inhibitors and CAR‐T cells improves the function of CAR‐T cells in TME and helps to overcome the resistance of cancer cells to treatment. Extensive transcriptional and epigenetic alterations and increased expression of inhibitory receptors lead to T cell exhaustion.^128^ Reducing CAR‐T cell depletion as well as enhancing the persistence of CAR‐T cells are necessary for better therapeutic outcomes. Increased expression of nuclear receptor subfamily 4A (NR4A) and thymocyte selection‐associated high mobility group box proteins 1 and 2 (TOX and TOX2) transcription factors can up‐regulate genes encoding repressor molecules to induce T cell exhaustion. It has been shown that CAR‐T cells knocking down the NR4A gene or lacking both TOX and TOX2 can reduce CAR‐T cell depletion in TME and have enhanced anticancer activity.^129^, ^130^

## 9 SUMMARY AND OUTLOOK

In recent years, a new class of experimental medicines known as CAR‐T cell therapy has emerged, showing promise in treating haematologic malignancies. Approximately 190 clinical studies are now being conducted in the field of immunotherapy. The outcomes of these investigations have been incorporated into phase I clinical trials. Functional evaluation of CAR‐T cells has advanced. With each study, a significant advancement in our comprehension of CAR‐T cell biology has been made. Despite the early clinical success of CAR‐T in the treatment of AML, research in this field is still in its early stages, and the high risk of relapse and variable levels of toxicity connected with CAR‐T therapy calls for more advancements in this technology. Examples include refining the CAR structure, studying new targets, modifying the immune milieu, creating universal CAR‐T treatment, using combination therapies, and other tactics. Our ongoing efforts are focused on improving targeted killing, combating targeted tumour antigen escape, decreasing the rate of recurrence, and minimizing harmful side effects. In the future, we think that more investigations by researchers will benefit tumour patients.

## AUTHOR CONTRIBUTIONS

**Chi Gao:** Data curation (equal); formal analysis (equal); investigation (equal); methodology (equal); resources (equal); writing – original draft (equal). **Xin Li:** Data curation (supporting); formal analysis (supporting); resources (supporting); writing – original draft (supporting). **Yao Xu:** Formal analysis (supporting); funding acquisition (equal); project administration (supporting); resources (supporting); writing – review and editing (equal). **Tongcun Zhang:** Formal analysis (equal); funding acquisition (lead); project administration (equal); supervision (equal); writing – review and editing (equal). **Haichuan Zhu:** Project administration (equal); resources (equal); supervision (equal); validation (equal); writing – review and editing (equal). **Di Yao:** Conceptualization (lead); data curation (lead); formal analysis (lead); funding acquisition (equal); investigation (lead); project administration (lead); resources (lead); supervision (lead); validation (lead); writing – original draft (lead); writing – review and editing (lead).

## FUNDING INFORMATION

This work was supported by a grant from the Wuhan Science and Technology Plan Project (2019030703011533), Wuhan East Lake High‐tech Zone ‘JieBangGuaShuai’ Project (2022KJB113), and Hubei Province Supporting Enterprise Technology Innovation Development Project (2021BAB12).

## CONFLICT OF INTEREST STATEMENT

The authors confirm that there are no conflicts of interest.

## Data Availability

The datasets used and/or analysed during the current study are available from the corresponding authoron reasonable request.
